# Words matter: a call for humanizing and respectful language to describe people who experience incarceration

**DOI:** 10.1186/s12914-018-0180-4

**Published:** 2018-11-16

**Authors:** Nguyen Toan Tran, Stéphanie Baggio, Angela Dawson, Éamonn O’Moore, Brie Williams, Precious Bedell, Olivier Simon, Willem Scholten, Laurent Getaz, Hans Wolff

**Affiliations:** 1 Division of Prison Health, Geneva University Hospitals and University of Geneva, Ch. du Petit-Bel-Air 2, CH-1225 Chêne-Bourg, Switzerland; 2 Australian Centre for Public and Population Health Research, Faculty of Health, University of Technology, PO Box 123, Sydney, NSW 2007 Australia; 3 Public Health England & UK Collaborating Centre, WHO Health in Prisons Programme, Premier House, 60 Caversham Road, Reading, RG1 7EB UK; 4 Division of Geriatrics, Criminal Justice & Health Program, University of California in San Francisco, 3333 California Street, San Francisco, CA 94118 USA; 5 Department of Psychiatry, School of Medicine & Dentistry, University of Rochester, 300 Crittenden Boulevard, Rochester, NY 14642 USA; 6 Psychiatry Department, Centre hospitalier universitaire de Lausanne, Av. Recordon 40, 1004 Lausanne, Switzerland; 7 Willem Scholten Consultancy, Wielsekade 64, 3411 AD Lopik, The Netherlands

**Keywords:** Health in prisons, Incarceration, Terminology, Access, Stigma, Discrimination, Harm reduction, Human rights

## Abstract

**Background:**

Words matter when describing people involved in the criminal justice system because language can have a significant impact upon health, wellbeing, and access to health information and services. However, terminology used in policies, programs, and research publications is often derogatory, stigmatizing, and dehumanizing.

**Discussion:**

In response, health experts from Europe, the United States, and Australia recommend that healthcare professionals, researchers, and policy makers working with people in detention follow key principles that foster constructive and humanizing language. These principles include: engage people and respect their preferences; use stigma-free and accurate language; prioritize individuals over their characteristics; and cultivate self-awareness. The article offers examples of problematic terms to be avoided because they do not convey respect for incarcerated people and propose preferred wording which requires contextualization to local language, culture, and environment.

**Conclusion:**

The use of respectful and appropriate language is a cornerstone of reducing harm and suffering when working with people involved in the criminal justice system; the use of stigmatizing and dehumanizing language must therefore come to an end.

## Background

Worldwide more than ten million people are held in penal institutions [1]. People who are incarcerated have greater physical and mental health needs than the general population [2] and can experience considerable stigma and discrimination that impact upon their access to health services [3]. To achieve health equity, this priority population needs adequately-resourced health services that are at least equivalent to those offered in the community [4]. Furthermore, increased research on health in prison is required to improve our understanding of factors that influence the health and wellbeing of incarcerated people so that health interventions best meet their needs [5]. Importantly, they must be engaged in the design and implementation of such services to ensure non-judgemental and stigma-free care.

Stigma can be enacted and reinforced through labelling. Such labelling can drive the stereotyping, prejudice, and discrimination of groups of people, such as individuals involved in the criminal justice system who are often denounced as being responsible for their incarceration. As a result, those in the criminal justice system are excluded from social and economic resources and services that ultimately affect their health and wellbeing [6]. Therefore, language used to describe individuals and populations, either respectful or stigmatizing, matters and shapes people’s views and understanding of past and present events, as well as future possibilities [7]. Individuals affected by the criminal justice system face multiple stigma as they may be arrested and incarcerated for their use of psychoactive substances, HIV status, mental health conditions, sexual orientation or behaviours, or irregular migratory situation [8]. Stereotyping can be engrained in cultural norms and values and in structures and systems, so much so that negative effects on marginalized groups may no longer be noticed, becoming part of routine behaviour. Such stigma and discrimination can affect the identities of incarcerated individuals, how they see themselves (from perceived stigma to internalized stigma [9]), and how society perceives ‘them’. Language used to describe incarcerated people, their life experience, behaviours, health risk factors, and medical conditions can therefore play an important role in supporting or undermining their health, wellbeing, and access to health information and services. However, terminology used to describe people affected by the criminal justice system in policies, programs, and research publications is often derogatory, stigmatizing, biased, and dehumanizing (e.g., *criminal, prisoner, felon, offender*, *drug addict*).

The use of respectful and person-centred language (also called ‘person-first language’) to describe individuals who are incarcerated, their characteristics, and experiences is fundamental to improving access to medication and services and minimizing discrimination [10]. Stigma-free language can positively influence media narratives, public opinion, and, above all, ensure that policy changes are inclusive of this priority population. The medical disciplines promote person-centred care that is culturally-sensitive and respectful of the ethical principles of autonomy, benevolence, and non-malevolence, which underpins quality of healthcare [11]. Respectful language is an integral part of this person-centred approach.

The World Health Organization (WHO) recommends in its 2013 style guide that language must not discriminate against, stereotype, or demean people on the basis of their age, physical or intellectual impairments, ethnicity, gender, sex or sexual orientation, although it does not address specifically problematic terminology used to describe people involved in the criminal justice system [12]. Despite these recommendations, the WHO landmark publication *Prison and Health* (2014) still uses language emphasizing imprisonment or criminality to describe incarcerated people (*prisoner, inmate, offender*) [13]. The Lancet series published in 2016 on *HIV and related infections in prisoners* widely used the term *prisoner* in its articles [14–18]. While the series title is crisp and effective in drawing attention to this major public health challenge, it is easy to associate the title with images of *prisoners infected* with HIV and other infections and posing a threat to society. Although *people incarcerated living with HIV and other infections* would be a mouthful alternative, it has the important merit of being person-centred with the aim of reducing stigma.

Efforts to promote a humanizing language have been undertaken in several contexts. These include sex and gender, sexuality, HIV [19], mental health [20], tuberculosis [21], and the use of psychoactive substances and controlled medicines [22]. The need to use humane language to describe individuals involved in the criminal justice system has recently gained momentum as evidenced by a ground-breaking policy document issued by the Scottish government in 2016 [23], followed by a letter to the editor in 2017 [24] and a commentary in 2018 [25] in peer-reviewed journals. Our paper extends this work and proposes four principles on the use of language to guide the way we speak, research, write, and communicate with and about the people affected by the criminal justice system. We highlight examples of stigmatizing and dehumanizing language and specific health conditions experienced by incarcerated people, before recommending alternative terminology that is person-centred, accurate, non-biased, respectful, and stigma-free.

## Guiding principles

### Engage people and respect their preferences

Table 1 compiles a list of commonly used terms to describe people involved in the criminal justice system and examples of health conditions and situations that can fuel negative stereotypes. We list problematic terms to be avoided and propose preferred wording. Policymakers, researchers, program managers, healthcare providers, criminal justice professionals, and custodial service commissioners should engage people who are currently or formerly incarcerated – such as two contributors to this article (SC, CG) and one of the co-authors (PB), respectively – and ask them about the language they prefer using to identify themselves. The Marshall Project in the USA [26] and User Voice in the UK [27] are examples of meaningful engagement with people involved in the criminal justice system. As terminology requires adaptation in local languages and cultures, each linguistic and professional community should be engaged in discussing and contextualizing these terms so that they are acceptable in the circumstances they are to be used.

**Table 1:** Examples of terminology to avoid (in alphabetical order), problems related to its use, and preferred wording to describe people who are incarcerated

Terminology to avoid	Problems	Preferred wording
Abuse; misuse	Judgmental; negates the fact that substance use disorders are a medical condition [22]; not conducive to fostering the trust and respect required when engaging with people who use psychoactive substances [19]	(Heavy) substance use; substance use disorder (Diagnostic and Statistical Manual of Mental Disorders – DSM-5); dependence syndrome (International Classification of Diseases – ICD-10)
Body-packer; drug mule; drug smuggler	Not person-centred language, judgmental	Person with body-packing, or with internal concealment of psychoactive substance [36]
Body-stuffer	Not person-centred language, judgmental	Person diagnosed with acute ingestion of psychoactive substance [36]
Correctional; offender; penitentiary; prison health services	Reinforces stereotypes, moralistic, ambiguous.	Health services in detention settings; healthcare in prison
Crazy; mental; insane; psycho; mentally ill; emotionally disturbed; demented	Not person-centred language, judgmental	Person living with a mental health condition; person living with dementia
Dungeon; hole	Derogatory, inaccurate, reinforces self-stigma	Solitary confinement
Drug user; abuser; addict; junkie; dependent	Not person-centred language, judgmental	Person with a substance use disorder; person with dependence syndrome; person who uses psychoactive substances
Ex-prisoner; ex-offender; ex-inmate; ex-felon; ex-con; criminal; thug; post-carceral	Not person-centred language, judgmental	Person who was in contact with, involved in, interacted with or experienced the criminal justice system; person with convictions; person who was formerly incarcerated
High(er)-risk group	Implies that the risk is contained within the group; can increase stigma and discrimination against the designated groups; membership of groups does not place individuals at risk, behaviours may [19]	Key populations; priority population; high-risk behaviour (e.g., sharing needles, condomless sex)
Hunger striker	Not person-centred language, judgmental	Person on hunger strike
Illegal immigrant; illegal; unlawful non-citizen; visa overstayer; undocumented alien	Not person-centred language, judgmental	Person who lacks resident documentation
Prisoner; inmate; felon; offender	Not person-centred language, judgmental	Person who is incarcerated; person who experience incarceration; person in detention/jail/prison; person living in detention/jail/prison; person involved in, or experiencing the criminal justice system
Prisoner-patient	Health staff care for patients, irrespective of their status	Patient; person in treatment
Prostitute or prostitution	Not person-centred language, judgmental [12]	Person involved in sex work, or in sale or trade of sexual services; sex worker
Probationer; parolee	Not person-centred language, judgmental	Person on parole; person on probation
Substitution therapy or opioid substitution therapy (OST)	Misleading: gives the impression to politicians, civil servants, and other lay people that this therapy is replacing ‘street drugs’ with ‘state drugs’; and therefore, this language counteracts availability of therapy [22]	Opioid agonist therapy (OAT); opioid agonist therapy for the treatment of substance use disorder; treatment [37]

### Use stigma-free and accurate language

People experiencing incarceration are family and community members, friends, students, teachers, or co-workers. Indeed, as stated by UNAIDS in 1996 at the United Nations Commission on Human Rights, ‘*Prisoners ****are**** the community. They come from the community, they return to it. Protection of prisoners is protection of our communities.’* However, people leaving imprisonment often face daunting barriers to reintegrate into their community and exercise their rights to housing, employment, health insurance coverage, education, voting or parenting, among others. Enabling them to re-enter society and preventing recidivism are primary objectives of detention. Terms that devalue, exclude, discriminate, stereotype, objectify, dehumanize, and reinforce a ‘criminal self-image’, such as *offender, criminal, felon*, *prisoner*, *convict,* should be avoided. *Inmate* should not be used as it is ambiguous and refers to people living in any institution, including psychiatric hospitals [28].

The language we use to conceptualize and talk about incarcerated people and their characteristics reflects our personal views and understanding, or, too often, our biases (conscious or unconscious) and lack of understanding. It also helps shape our own and others’ attitudes about people involved in the criminal justice system and the way we grant or limit (at times unknowingly) their access to services. Therefore, defining people by the crime for which they were convicted (e.g., *drug dealer, murderer, rapist, sex offender, paedophile*) or by their legal status (e.g., *illegal immigrant*), and using moralistic language regarding substance use (e.g., *drug abuser)* or work (e.g., *prostitute)* is not helpful in supporting respectful interaction.

Use of the term *correctional*, which has been common since the 1950s in North America to describe the criminal justice system and related institutions (e.g., *correctional health services*), should be re-examined: it is linked to the sombre history of forced correctional labour camps [29], can be construed as moralistic, and is underpinned by the concept of ‘deviant’ behaviours to be corrected (while many people instead require treatment and care, rather than ‘behaviour correction’ such as for substance use disorders or mental health conditions). We should also reconsider the use of the term *penitentiary* (from *penitens* in Latin, meaning regretting or repenting) due to the strong religious connotation: ‘God’s forgiveness’ requires *penitence* and implies a sad and humble regret for one’s sins or wrongdoing.

Double denomination, such as *prisoner-patient,* should not be used in health service guidelines or by healthcare professionals [30]. The term puts the detention status of people before their needs for medical attention. It emphasizes the dual loyalty confusion for health professionals: one that often obfuscates the provider’s primary relationship (towards healthcare principles and ethics or towards the prison authorities) [31]. Healthcare providers, even if they are directly employed by prison authorities, must first attend to their patients as patients and act independently of prison or judicial authorities [32].

### Prioritize the individual

Incarceration is often perceived as the worst experience of one’s life. However, individuals in detention are not defined only by the experience. Even when restricted in their freedom of movement, people must be given the resources to keep living with dignity and respect. Healthcare professionals have learned not to label patients by their medical diagnosis (e.g., we use *person with body packing or with internal concealment of psychoactive substance* instead of *body packer; person on hunger strike* instead of *hunger striker*). Likewise, we recommend placing individuals at the centre, and their characteristics or medical conditions second in the description. Therefore, the use of person-centred language should be preferred to describe what people have or the circumstances in which they live, which in the end should not define who they are and how we treat them. For instance: *person who is incarcerated* or *living in detention/prison/jail* (instead of *prisoner)* or *person living with HIV* (instead of *HIV-infected patient*) emphasize the fact that individuals are not powerless and can continue to live with dignity despite their environment or condition; *person formally incarcerated, person with convictions* (instead of *ex-con*) factually describes people in a specific phase of their life.

### Cultivate self-awareness

Professionals working with people who are incarcerated should be conscious of the language they use as it can convey powerful images and meanings. They should favour in their clinical practice, policy, and research the use of humane and constructive language that promotes respect, dignity, understanding, and positive outlooks, and should encourage colleagues, friends, and their community to do so. Likewise, recognizing their influence in positively shaping public opinion, we call upon scientific journals, the media, governments, national and international organizations, including the legal community, to strive to adopt language that respects the dignity of people involved in the criminal justice system. While some people may not use preferred terminology, it is important for professionals of all sectors to develop cultural humility and self-reflection [33], be mindful, and refrain from repeating negative terms that discriminate, devalue, and perpetuate harmful stereotypes and power imbalances. Values clarification workshops for healthcare (and non-healthcare) professionals and researchers working with people involved in the criminal justice system could be transformative in clarifying values and changing attitudes to improve interactions with others [34], as may interventions to transform self-stigma and build the coping skills of individuals incarcerated, their families, and children [35]. Such interventions have the power of challenging prejudices, stigma, and self-stigma by increasing an individual’s awareness of values that may have a bearing on decisions and actions in their lives. Values clarification can therefore enhance our understanding of the complex sociocultural, psychological, and behavioral determinants of incarceration, redirect personal values, and address potential barriers to change the use of inappropriate language (e.g., through supportive supervision of staff working with incarcerated people). These actions can work to assist professionals to prioritize the use of terminology that adheres to our professional mandate: caring for people and supporting them in their journey of recovery and reintegration into society. Our use of language must promote such processes.

## Conclusion

Respectful language is a cornerstone of reducing harm and suffering. Healthcare professionals, researchers, and policy makers working with people involved in the criminal justice system can be guided by key principles that foster constructive and humanizing language: engage people and respect their preferences, use stigma-free and accurate language, prioritize individuals over their characteristics, and cultivate self-awareness. Problematic terms must be avoided because they do not convey respect. However, the preferred wording we propose is far from being universal: it requires contextualization to local language and socio-cultural environment. Tension can also arise when trying to use respectful language and at the same time be concise and efficient, especially in policy documents, guidelines, and scientific publications. This requires a pragmatic approach, but one that should not sacrifice the promotion and use of stigma-free and more accurate language. We call upon all professionals to be self-reflective and mindful that names can hurt and that appropriate language to describe people experiencing incarceration can reduce harm and enhance health and wellbeing. We must therefore strive to develop a suitable vocabulary and communication style that embody respect, dignity, and humanity for all people – free or incarcerated.

## Abbreviation

WHO: World Health Organization
